# Synthesis of Novel Pyrimethanil Grafted Chitosan Derivatives with Enhanced Antifungal Activity

**DOI:** 10.1155/2016/8196960

**Published:** 2016-07-27

**Authors:** Yan Li, Song Liu, Yukun Qin, Ronge Xing, Xiaolin Chen, Kecheng Li, Pengcheng Li

**Affiliations:** ^1^Institute of Oceanology, Chinese Academy of Sciences, No. 7 Nanhai Road, Qingdao 266071, China; ^2^University of Chinese Academy of Sciences, Beijing 100049, China; ^3^Key Laboratory of Experimental Marine Biology, Institute of Oceanology, Chinese Academy of Sciences, No. 7 Nanhai Road, Qingdao 266071, China

## Abstract

In this study, three pyrimethanil grafted chitosan (PML-g-CS) derivatives were obtained. The structures of the conjugates were confirmed by FT-IR, ^1^H NMR, and EA. The grafting ratios were measured by HPLC. Antifungal properties of pyrimethanil grafted chitosan (PML-g-CS) derivatives against the plant pathogenic fungi* Rhizoctonia solani* and* Gibberella zeae *were investigated at concentrations of 100, 200, and 400 mg/L. The PML-g-CS derivatives showed enhanced antifungal activity in comparison with chitosan. The PML-g-CS-1 showed the best antifungal activity against* R. solani*, whose antifungal index was 58.32%. The PML-g-CS-2 showed the best antifungal activity against* G. zeae*, whose antifungal index was 53.48%. The conjugation of chitosan and pyrimethanil showed synergistic effect. The PML-g-CS derivatives we developed showed potential for further study and application in crop protection.

## 1. Introduction

Chitosan (CS), a kind of natural cationic polysaccharides, consists of *β*-1,4-linked glucosamine with various N-acetyl glucosamine residues [1]. It is naturally renewable and exhibits a lot of special properties like biocompatibility, biodegradability, and bioactivity. Due to its special chemical structure and characteristics, chitosan is widely used in many areas such as food industry, medical cares, and agriculture [2]. Particularly, it was proved to have a broad-spectrum antifungal activity against a variety of fungi. Allan and Hadwiger found that, of the 46 tested fungi, chitosan had antifungal activity against 32 isolates [3]. Bautista-Baños et al. described the fungicidal activity of chitosan on* Colletotrichum gloeosporioides* in vitro stating that 3% chitosan can completely inhibit its growth [4]. However, chitosan also shows some demerits for its further application; the most important limit is that its antifungal effect is weaker than many commercial fungicides. Thus, there have been a lot of methods to enhance its antifungal effect [5]. One of the approaches is the chemical modification of chitosan. A wide range of bioactive groups were combined to the chitosan molecular structure to achieve different chitosan derivatives such as carboxymethyl chitosan [6], sulfate chitosan [7], chitosan quaternary ammonium salt [8], and hydroxypropyl chitosan [9].

Recently, the synthesis and property study of chitosan conjugates has received increased attention. Different kinds of molecules are grafted onto chitosan chains by chemical bond [10]. The complexation of chitosan and metal ions enhanced its antibacteria activity [11, 12]. The conjugation by grafting of antioxidant molecules onto chitosan improves the antioxidant activity [13]. However, little attention has been paid to the improvement of chitosan antifungal activity by the conjugation of antifungal molecules [14, 15].

Pyrimethanil (PML) is a kind of traditional fungicide widely used in agriculture [16]. Its commercial product name is “Scale”. It occupies great share in commercial fungicide market due to its high-efficiency and special mechanism [17]. Pyrimethanil has excellent antifungal effect on grey mould of grapes and strawberries. It inhibits the growth of* Monilia laxa* and* Drepanopeziza ribis *observably. The antifungal mechanism of pyrimethanil is to inhibit the secretion of fungi infecting enzyme. It has both protecting and treating function.

In order to improve the antifungal activity of chitosan, we did our research on the conjugation of chitosan and pyrimethanil technical material. In our study, pyrimethanil was grafted onto the chitosan polymer chains for the first time. The synthesized pyrimethanil grafted chitosan derivatives were characterized by Fourier transform infrared (FT-IR), nuclear magnetic resonance (NMR), and element analysis (EA) to confirm the conjugation. The grafting ratios were detected by HPLC. The antifungal activities of the compounds were also determined.

## 2. Experiment

### 2.1. Materials and Reagents

Chitosan (86.3% degree of deacetylation) was purchased from Qingdao Yunzhou Biochemical Corp. Pyrimethanil technical material (purity higher than 96%) was purchased from Yantai Kechuang Chemical Corp. Chloroacetyl chloride was purchased from Sinopharm Chemical Reagent Co., Ltd. Chloropropionyl chloride and 4-(chloromethyl) benzoyl chloride were purchased from Aladdin Chemical Reagent Co., Ltd. Isopropanol, dichloromethane, trichloromethane, potassium carbonate, glacial acetic acid, methanol, sodium hydroxide, and tween 80 were purchased from Sinopharm Chemical Reagent Co., Ltd. and were all of analytical grade. Methanol, sodium acetate, and acetic acid for HPLC analysis were purchased from Merck Drugs & Biotechnology and were of chromatographic grade.

### 2.2. Analytical Methods

Fourier transform infrared (FT-IR) spectra range within the 4000–400 cm^−1^ regions on a Thermo Scientific Nicolet iS10 FT-IR spectrometer with attenuated total reflection intelligent components. ^1^H NMR (nuclear magnetic resonance) spectra were investigated on a JEOL JNM-ECP600 spectrometer; solvents were CD_3_COOD and D_2_O. The elemental analysis (C, H, and N) was performed using a Vario EL-III elemental analyzer. The percentages of carbon, nitrogen, and hydrogen were estimated. The grafting ratios of pyrimethanil grafted chitosan derivatives were determined by an Agilent 1260 HPLC (Agilent Technologies, USA) equipped with a UV-detector. Chromatography was performed on C18 reversed-phase column.

### 2.3. Synthesis of Pyrimethanil Grafted Chitosan Derivatives

The synthesis was carried out by two steps. For the first step, pyrimethanil technical material was modified by chloroacetyl chloride, chloropropionyl chloride, and 4-(chloromethyl) benzoyl chloride to achieve a series of intermediates for the following reaction. The second step was the synthesis of pyrimethanil grafted chitosan derivatives [18].

In the first step, pyrimethanil (4 g) was dissolved in dichloromethane (l80 mL). After that, 2 times the equivalent amount of chloroacetyl chloride (chloropropionyl chloride or 4-(chloromethyl) benzoyl chloride) was added into the dichloromethane in drops in an ice bath. 1.2 times of equivalent amount of potassium carbonate was also added into the reaction system. The end of reaction was tested by thin layer chromatography (TLC). When the reaction was over, the reaction mixture was added into water (50 mL) to remove extra acyl chloride. The reaction mixture was poured into a separating funnel. The organic phase at the lower layer was collected and evaporated to dryness to achieve the intermediates.

In the second step, purified chitosan (3 g) was dispersed in trichloromethane (50 mL). After 30 minutes of magnetic stirring at room temperature, 12 mL of aqueous NaOH (10 M) was added to the suspension and the alkalization lasted for 4 hours. The reaction proceeded to 4 h at temperature of 50°C and the solid product was then filtered, suspended in 150 mL of methanol, and neutralized with glacial acetic acid. The product was extensively washed with 100% methanol and dried at room temperature. Finally, the pyrimethanil grafted chitosan derivatives were obtained.

### 2.4. Determination of Weight Average Molecular Weight of Pyrimethanil Grafted Chitosan Derivatives

The weight average molecular weight (Mw) of chitosan and PML-g-CS derivatives were measured by gel permeation chromatography using Agilent 1260 HPLC (Agilent Technologies, USA) equipped with a refractive index detector. Chromatography was performed on TSK G5000-PWXL column, using 0.2 M CH_3_COOH/0.1 M CH_3_COONa aqueous solution as mobile phases at a flow rate of 1.0 mL/min with column temperature at 30°C. The sample concentration was 0.4% (w/v). The standards used to calibrate the column were dextrans samples with Mw of 1,300,000, 670,000, 270,000, 80,000, 50,000, and 25,000 Da (Sigma, USA).

### 2.5. Determination of Grafting Ratios of Pyrimethanil Grafted Chitosan Derivatives

0.1 g PML-g-CS was added into methanol solution (100 mL, pH 4.0). Hydrolysis lasted 2 hours to extricate all the pyrimethanil from the conjugates. Then, the solution was diluted to 10 times for detection. The determination of grafting ratio of PML-g-CS derivatives was measured by an Agilent 1260 HPLC (Agilent Technologies, USA) equipped with a UV-detector. Chromatography was performed on C18 reversed-phase column, using H_2_O/methanol (15 : 85) solution as mobile phases at a flow rate of 1.0 mL/min with column temperature at 30°C. The testing wave length was set to 220 nm [19]. The standards used to calibrate the results were pyrimethanil dissolved in methanol with a series concentration of 0.01, 0.02, 0.04, 0.08, and 0.1 mg/mL. The grafting ratios were calculated based on the standard curve.

### 2.6. Antifungal Activity Assay

Antifungal assays against* Rhizoctonia solani* CGMCC 3.28 and* Gibberella zeae *CGMCC3.42 were evaluated in vitro according to the agar medium method described in literature [20]. Chitosan and PML-g-CS derivatives were dissolved in 0.5% (v/v) acetic acid at an original concentration of 1% (w/v). The solutions were mixed with sterile molten potato dextrose agar (PDA) to obtain final concentrations of 100 mg/L, 200 mg/L, and 400 mg/L.

The antifungal index was calculated by the following formula:

(1) $$\begin{array}{c} \text{Antifungal Index }\left(\;\text{\%}\;\right)=\frac{D_{\text{blank}}-D_{\text{average}}}{D_{\text{blank}}-D_{\text{fungus}}}\times 100. \end{array}$$

*D*_blank_ referred to the diameter of the blank plate without fungus inoculated. *D*_average_ referred to the average diameter of the fungal colony. *D*_fungus_ referred to the diameter of the inoculated fungal disk.

### 2.7. Statistical Analysis

Antifungal bioassay experiments were performed in triplicate. The data were analyzed with Origin, version 8.0 (Origin Lab Corp., Northampton, MA), and all the results were expressed as the mean ± SD. Results with *P* < 0.05 were considered to be statistically significant.

## 3. Results and Discussion 

### 3.1. Preparation of Reaction Intermediates and Pyrimethanil Grafted Chitosan Derivatives

**Figure 1:**
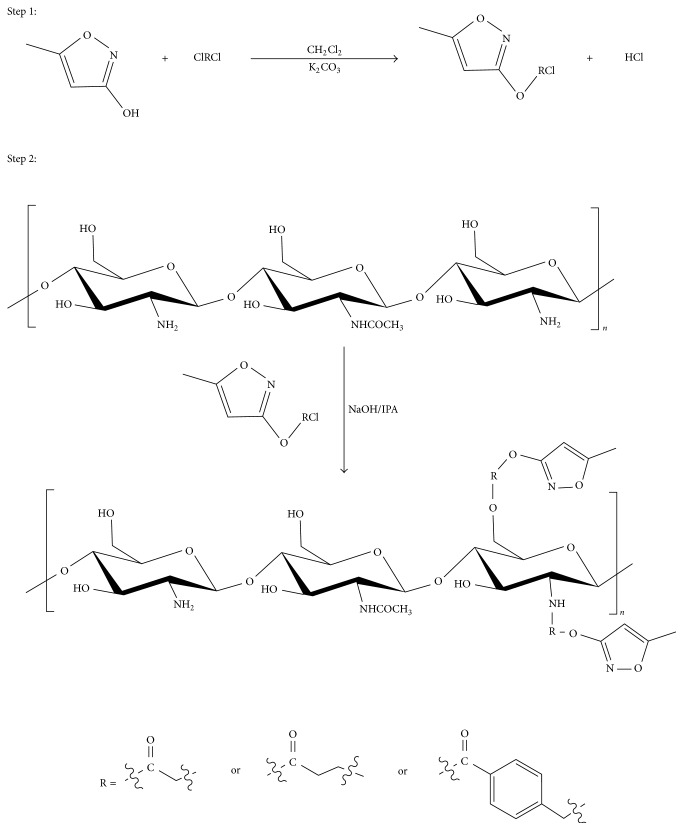
The reaction process for the PML-g-CS derivatives.

In this study, pyrimethanil was grafted onto chitosan using three kinds of acyl chlorides as linkers. The grafting reaction was carried out by two steps: the synthesis of intermediates and the synthesis of PML-g-CS derivatives. The reaction process for the conjugation of pyrimethanil grafted chitosan derivatives was proposed in Figure 1. For the first step, three kinds of reaction intermediates were synthesized by mixing pyrimethanil and acyl chlorides in dichloromethane. The acid-binding agent was potassium carbonate. The end of reaction was tested by thin layer chromatography (TLC). The developing solvent of the TLC process was methanol and petroleum ether (v : v = 1 : 5). For the second step, PML-g-CS derivatives were obtained. We named the conjugates whose linkers were chloroacetyl chloride, chloropropionyl chloride, and 4-(chloromethyl) benzoyl chloride as PML-g-CS-1, PML-g-CS-2, and PML-g-CS-3, respectively.

The graft ratios measured by HPLC for three PML-g-CS derivatives were 8.13%, 6.54%, and 5.05%. The standard curve was* y* = 106179*x *− 1116.2, and* R* ^2^ = 0.992.

### 3.2. Characterization of Pyrimethanil Grafted Chitosan Derivatives

#### 3.2.1. FT-IR Spectra

**Figure 2:**
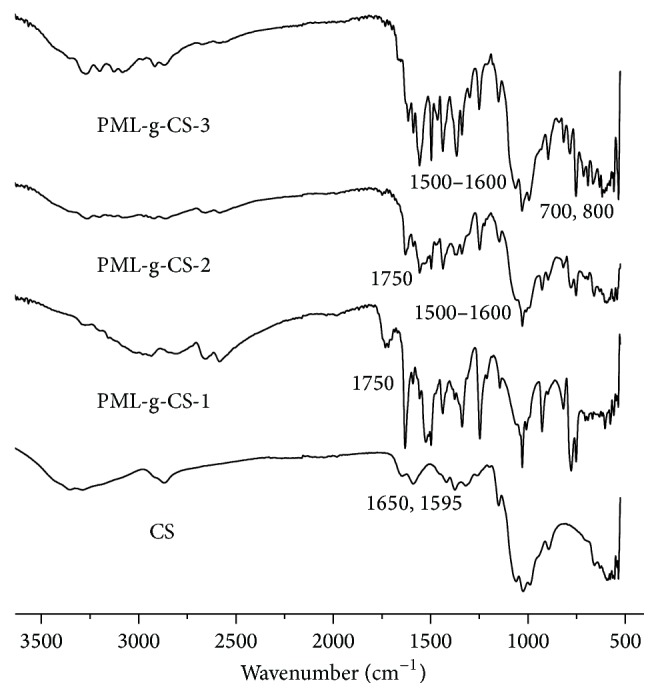
FT-IR spectra of chitosan and PML-g-CS derivatives.

Figure 2 showed FT-IR spectra of chitosan, PML-g-CS-1, PML-g-CS-2, and PML-g-CS-3. For chitosan, the broad band around 3384 cm^−1^ was attributed to OH and NH stretching vibration. The weak band at 2879 cm^−1^ was the characteristic absorbance peak of CH. The band at 1595 cm^−1^ was assigned to the N-H bending of the primary amine. The bands at 1650, 1550, and 1320 cm^−1^ were attributed to the C-O stretching, N-H bending, and C-N stretching of the residual N-acetyl groups, respectively. For pyrimethanil, the typical absorption peaks between 1500 and 1600 cm^−1^ were due to the C-N band and benzene ring [21]. For PML-g-CS-1 and PML-g-CS-2, the obvious absorption peak at 1750 cm^−1^ was attributed to bending vibration of C-H which belongs to the previous acyl chloride structure. For PML-g-CS-3, new bands at 700 cm^−1^ and 800 cm^−1^ were observed. Those C-H bands' absorption peaks indicated a monosubstituted benzene ring and a p-substituted benzene ring, which fitted the theoretical structure of PML-g-CS-3. All of the above results exhibited that pyrimethanil grafted chitosan derivatives had been successfully prepared.

#### 3.2.2. ^1^H NMR Spectra

**Figure 3:**
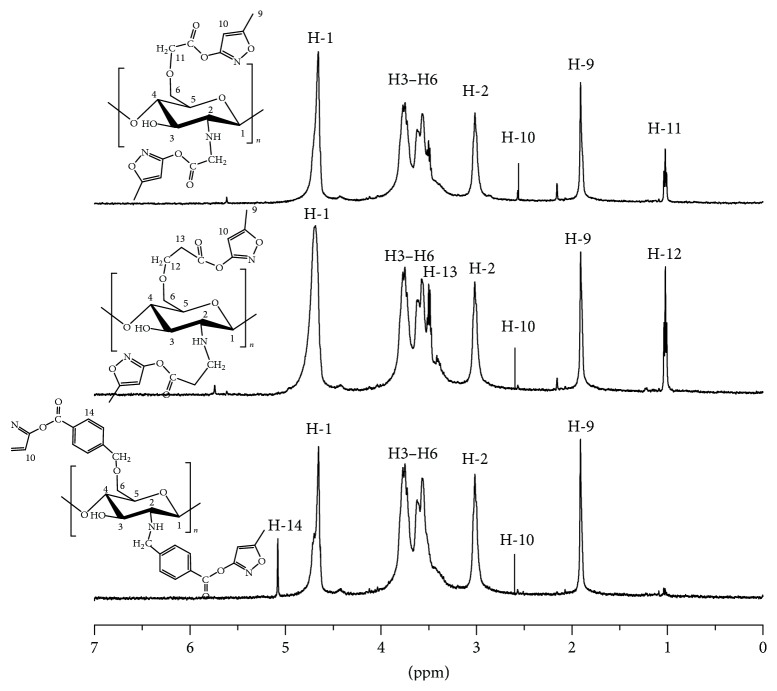
^1^H-NMR spectra of chitosan and PML-g-CS derivatives.

The ^1^H NMR spectra of chitosan, PML-g-CS-1, PML-g-CS-2, and PML-g-CS-3 were shown in Figure 3. As shown in Figure 3, a series of peaks belonging to chitosan was detected: H-1 signal was the single peak at 4.4 ppm, H-2 peak was at 2.9 ppm, and peaks of H-3 to H-6 were the multiple peaks from 3.3 ppm to 3.7 ppm. For PML-g-CS derivatives, the characteristic absorption peaks of pyrimidine group were at 1.8 ppm and 5.6 ppm. The absorption peaks at 1.0 ppm and 3.3 ppm were attributed to chloroacetyl chloride and chloropropionyl chloride parts as linkers in PML-g-CS-1 and PML-g-CS-2, respectively. The absorption peaks of benzene protons of PML-g-CS-3 at 5.1 ppm were observed. All the characteristic absorption peaks of each derivative were marked in Figure 3 separately. Consistent with the FT-IR results above, the structure of PML-g-CS derivatives was further confirmed by ^1^H-NMR.

#### 3.2.3. Gel Permeation Chromatography

Taking into account that the antifungal activity of chitosan is dependent upon the molecular weight, we calculated the weight average molecular weight of chitosan and PML-g-CS derivatives by GPC. The results were shown in Table 1. The degree of dispersity was between 1.1 and 1.4, showing that the results were measured validly. The GPC results showed that the Mw of chitosan and PML-g-CS derivatives did not change in statistics. In such condition, the average molecular weight did not have an impact on the antifungal activity results.

#### 3.2.4. Elemental Analysis

Elemental analysis results of PML-g-CS derivatives are shown in Table 2. The degree of substitution (DS) of PML-g-CS derivatives was calculated on the basis of the percentage. As shown in Table 2, the DS of PML-g-CS derivatives were 8.45%, 6.72%, and 5.18%, which matched the results calculated by the HPLC method.

### 3.3. Antifungal Activity of Pyrimethanil Grafted Chitosan Derivatives

Antifungal properties of pyrimethanil grafted chitosan (PML-g-CS) derivatives against the plant pathogenic fungi* R. solani* and* G. zeae* were investigated at concentrations of 100, 200, and 400 mg/L.

**Figure 4:**
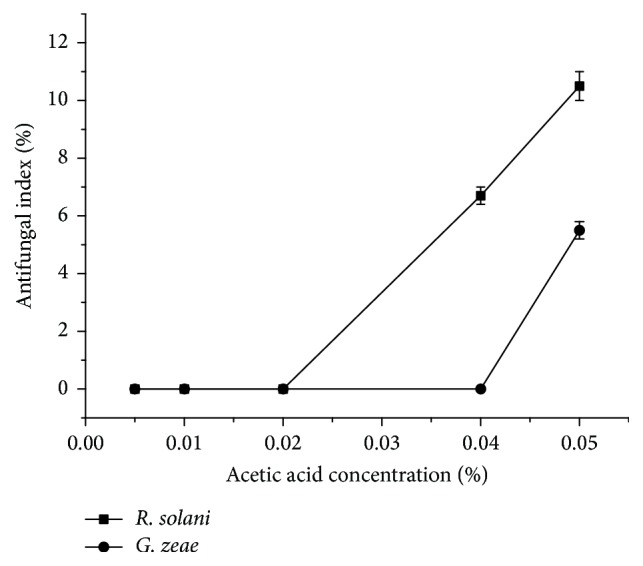
Inhibitory effect of different concentrations of acetic acid aqueous solution.

The antifungal activities of chitosan and chitosan derivatives are influenced by various factors such as molecular weight, degree of deacetylation, sample concentration, and pH value [22]. In this study, chitosan and pyrimethanil grafted chitosan (PML-g-CS) derivatives were dissolved in 0.5% (v/v) acetic acid at an initial concentration of 1% (w/v). The sample solutions were all diluted with sterile molten potato dextrose agar (PDA). The addition of the acetic acid might contribute to the antifungal effect. So the inhibitory activity of different concentrations of acetic acid aqueous solution against* R. solani* and* G. zeae *was evaluated. The testing concentrations were set corresponding to the investigated concentrations of samples. As shown in Figure 4, the inhibitory activity of acetic acid on the selected fungi was influenced by the concentrations. At concentrations lower than 0.02% (corresponding to 400 mg/L of the sample), acetic acid aqueous solution had no inhibitory effect on* R. solani* and* G. zeae*. The results showed that, at concentrations ranging from 100 to 400 mg/L, the diluted acetic acid had no antifungal activity. When the concentrations were higher than 0.02%, acetic acid would inhibit the growth of* R. solani* and* G. zeae*. It was shown that 0.05% acetic acid aqueous solution inhibited growth of* R. solani *at 10.5%.

**Figure 5:**
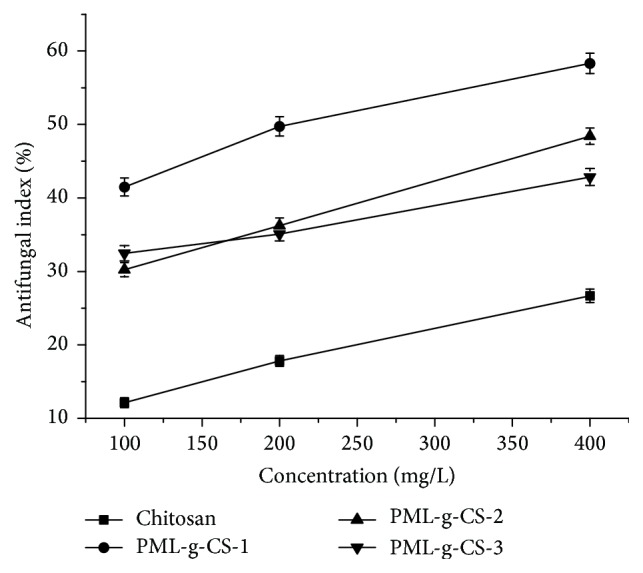
Antifungal activity of PML-g-CS derivatives against* R. solani*.

*R. solani* is a pathogen which causes diseases on paddies, peanuts, sesame, and so on [23]. As shown in Figure 5, all PML-g-CS derivatives exhibited much better antifungal activity compared with the original chitosan. The antifungal activity was enhanced with the increasing of concentration. The antifungal indexes of PML-g-CS derivatives ranged from 42.85% to 58.32% at 400 mg/L, while the indexes of chitosan only reached by 26.68%. The inhibitory activity followed a sequence of PML-g-CS-1 > PML-g-CS-2 > PML-g-CS-3 > chitosan. Different linkers also impacted the antifungal activity. The chloroacetyl chloride as linker was better than chloropropionyl chloride and 4-(chloromethyl) benzoyl chloride. This may be due to two reasons. One reason is that the smaller linker led to easier combination with the action sites. The other is that the grafting ratio is the highest when using chloroacetyl chloride as linker. Therefore, more pyrimethanil was on the polymer chain.

*G. zeae *is a pathogen mainly infecting gramineous plants, such as corn and wheat [24]. Table 2 represents the antifungal results of the PML-g-CS derivatives against* G. zeae*. Compared with chitosan, antifungal activity of PML-g-CS derivatives was enhanced. But the degree of improvement was not as obvious as the effect against* R. solani*. The antifungal activity of the original chitosan against* G. zeae *was stronger than* R. solani*, while the antifungal activity of PML-g-CS derivatives against* G. zeae* was weaker than* R. solani*. The antifungal index of PML-g-CS-3 at 400 mg/L was 27.9%, which was lower than the index of chitosan at the same concentration (28.72%). The results indicated that the modification by grafting pyrimethanil onto chitosan did not obviously enhance the antifungal activity against* G. zeae*.* G. zeae *was not sensitive to the new compounds.

By analyzing the abovementioned results, it was found that the DS indexes greatly influenced the antifungal activity of all PML-g-CS derivatives. The DS indexes of three derivatives changed with different linkers used in the synthesis process. The higher DS meant more pyrimethanil was grafted to the chitosan structure. What is more, the grafted pyrimethanil group contributed a lot to the improvement of antifungal activity of the PML-g-CS derivatives. The DS followed a sequence of PML-g-CS-1 > PML-g-CS-2 > PML-g-CS-3; meanwhile, the antifungal activity had the same results against both* R. solani *and* G. zeae.*

As shown in Table 3, the antifungal activity of pyrimethanil against* R. solani* and* G. zeae *was much stronger than the antifungal activity of PML-g-CS derivatives. At concentrations higher than 100 mg/L, the inhibition indexes of pyrimethanil technical material against* R. solani* and* G. zeae *were both 100%, while the highest inhibition effect of PML-g-CS derivatives was 58.32% and 53.48%.

When the concentrations of PML-g-CS derivatives were 100, 200, and 400 mg/L, considering the grafting ratios, the content of pyrimethanil was about 8, 16, and 32 mg/L, respectively. At the equivalent concentration, the antifungal activity of PML-g-CS derivatives was stronger than pyrimethanil technical material. This meant that the conjugation of chitosan and pyrimethanil showed synergistic effect. It was an efficient method to enhance the antifungal activity of chitosan.

Based on the bioassay results above, it was found that pyrimethanil grafted chitosan (PML-g-CS) derivatives had enhanced antifungal activity in comparison with chitosan. The PML-g-CS-1 derivative showed the best effect of three. The graft of pyrimethanil onto chitosan chain could improve the antifungal activity of chitosan in general. Further research may force the increase of antifungal activity to achieve a more economical product.

## 4. Conclusion

The preparation, characterization, and antifungal activity of pyrimethanil grafted chitosan (PML-g-CS) derivatives were investigated in the present study. Results suggested that the pyrimethanil could be grafted onto chitosan by using different acyl chlorides as linkers. In addition, the conjugation was confirmed by FT-IR and EA. Antifungal assays of chitosan and PML-g-CS derivatives against* R. solani* and* G. zeae* were evaluated in vitro. Statistical analysis also indicated that antifungal activity of chitosan was enhanced by grafting with pyrimethanil. Our data provided a novel approach for the modification of chitosan and indicated pyrimethanil grafted chitosan derivatives could be explored as promising antifungal agents.

## Figures and Tables

**Table 1:** The weight average molecular weight of chitosan and pyrimethanil grafted chitosan derivatives.

Samples	Mw (kDa)	Degree of dispersity
Chitosan	1058.6	1.28
PML-g-CS-1	1057.5	1.35
PML-g-CS-2	1053.4	1.17
PML-g-CS-3	1055.2	1.32

**Table 2:** Elemental analysis results and degree of substitution of chitosan and pyrimethanil grafted chitosan derivatives.

Samples	Elemental analysis (%)			Degree of substitution (%)
	C	N	H	
Chitosan	40.05	7.29	6.41	—
PML-g-CS-1	40.16	8.06	6.24	8.45
PML-g-CS-2	40.28	7.96	6.22	6.72
PML-g-CS-3	40.10	7.75	6.13	5.18

**Table 3:** Antifungal activity of chitosan and pyrimethanil grafted chitosan derivatives at different concentrations.

Samples	Concentrations (mg/L)	Antifungal index (%)	
		*R. solani*	*G. zeae*
Chitosan	100	12.13	15.55
	200	17.82	20.8
	400	26.68	28.72

PML-g-CS-1	100	41.49	27.58
	200	49.75	36.5
	400	58.32	48.9

PML-g-CS-2	100	30.23	37.26
	200	36.23	44.59
	400	48.4	53.48

PML-g-CS-3	100	32.47	25.85
	200	35.09	34.5
	400	42.85	38.9

Pyrimethanil	8	29.45	27.9
	16	33.75	32.35
	32	38.2	37.15
	100	100	100
	200	100	100
	400	100	100
